# Do anticoagulants affect outcomes of hip fracture surgery? A cross-sectional analysis

**DOI:** 10.1007/s00402-019-03240-5

**Published:** 2019-09-21

**Authors:** Caroline Hoerlyck, Terence Ong, Merete Gregersen, Else Marie Damsgaard, Lars Borris, Jac Kie Chia, Ying Yi Wendy Yap, Namal Weerasuriya, Opinder Sahota

**Affiliations:** 1 Aarhus University, Aarhus, Denmark; 2 Department for Healthcare of Older People, Queens Medical Centre, Nottingham University Hospitals NHS Trust, Derby Road, Nottingham, NG7 2UH UK; 3 Division of Rehabilitation and Ageing, School of Medicine, University of Nottingham, Nottingham, UK; 4 Department of Clinical Medicine, Geriatric Section, Aarhus University Hospital, Aarhus, Denmark; 5 Department of Clinical Medicine, Orthopaedic Section, Aarhus University Hospital, Aarhus, Denmark

**Keywords:** Anticoagulants, Warfarin, DOAC, Hip fractures, Trauma

## Abstract

**Introduction:**

The management of patients with a hip fracture is affected by the use of oral anticoagulants. A cross-sectional analysis was undertaken to investigate health outcome differences in those anticoagulated compared to those not anticoagulated.

**Methods:**

Patients aged 50 years and over presenting to a large university hospital with hip fractures were identified from the service registry. Patient characteristics and health outcomes between those not anticoagulated were compared with those anticoagulated (warfarin and direct oral anticoagulants, DOAC).

**Results:**

200/2307 (9%) patients were anticoagulated. 84% were on warfarin, and the rest a DOAC. Compared to those anticoagulated, there was a higher prevalence of dementia (25% vs. 18%, *p* = 0.02) and a lower prevalence of cardiovascular disease (54% vs. 78%, *p* < 0.01), atrial fibrillation (10% vs. 82%, *p* < 0.01), and polypharmacy (55% vs. 76%, *p* < 0.01). Renal function was lower in the anticoagulated group. Time to operation for those not anticoagulated and anticoagulated was a median (IQR) of 25 (15) and 27 (18) hours. There was no difference in blood transfusion and hospital mortality. Postoperative complications were similar except a higher rate of renal failure (14% vs. 19%, *p* = 0.04) and heart failure (1% vs. 5%, *p* < 0.01), and a longer length of stay [median (IQR): 14 (10) vs. 16 (12) days] in the anticoagulated group. This was no longer significant after adjustment of confounders.

**Conclusion:**

There was no statistically significant difference in health outcomes between those anticoagulated and those not after adjusting for patient characteristics. It was feasible to avoid significant delay in hip fracture surgery in those anticoagulated.

## Introduction

A hip fracture is a devastating injury that carries with its poor health outcomes. The overall number of hip fractures is expected to go up with the expected increase in number of older people [1]. Consequently, its negative socioeconomic impact worldwide will also increase significantly [2]. Surgery conducted within 48 h has been shown to improve these outcomes [3–12]. An operative wait beyond this time increases the risk of developing post-fracture complications [11, 12] and is associated with a 30-day and one-year mortality of 40% and 30%, respectively [9]. A recent study has reported that even a waiting time of more than 24 h was associated with worse outcomes, suggesting that this may be the threshold where adverse outcomes starts to increase [13]. Hence, minimising surgical delay appears to be an important factor in improving patient’s prognosis from their hip fracture.

Anticoagulated patients presenting with a hip fracture have been associated with delays in surgical fixation [14–18]. The potential need for reversal and fear of bleeding has been postulated as reasons for this delay. Clinical data have reported that in recent years there has been a steady increase in the number of people prescribed anticoagulation medication [19]. Therefore, it is anticipated that there will be more people prescribed oral anticoagulation presenting with hip fractures. There remains uncertainty of how best to manage these patients and their varied anticoagulation, of either a vitamin K antagonist, e.g. warfarin, or one of the new direct oral anticoagulants (DOACs). The aim of this study was to compare patient characteristics and outcomes of those on oral anticoagulation and those not, presenting to hospital with a hip fracture.

## Methods

A cross-sectional analysis was undertaken from the hip fracture registry of a large university hospital, Queens Medical Centre, Nottingham, UK. Characteristics and outcomes between patients presenting with hip fractures on oral anticoagulants (warfarin and DOAC) and those not anticoagulated were compared. The hospital’s hip fracture registry gathers information on all patients admitted to hospital with a hip fracture based on documentation made in clinical notes by the respective patients’ team. The data are sourced by audit data clerical staff from relevant clinical notes and entered electronically onto a hospital-secured electronic database. Patients aged 50 years and over admitted from 1 August 2013 to 29 July 2016 were identified from this registry. Patients were excluded if the fracture was attributed to a high impact trauma, secondary to cancer or if the fracture was non-operatively managed.

In this centre, patients on warfarin pre-surgery will receive vitamin K (phytomenadione) to reverse its anticoagulation effect to an International Normalised Ratio (INR) of less than 1.5. Those anticoagulated with a DOAC are operated > 24 h after the last dose was taken. Anticoagulation is restarted day 1 postoperatively unless the bleeding risk is deemed high by the attending clinician. For patients restarting warfarin, low-molecular weight heparin (enoxaparin) is given for prophylaxis until the INR is greater than 2.0. Red blood cell transfusion was indicated if haemoglobin level was either less 8 g/dL or less than 10 g/dL with symptomatic anaemia.

Those on warfarin and DOACs were grouped together due to a small number of patients presenting on a DOAC during the analysis period. DOACs that were likely prescribed during the study period were rivaroxaban, apixaban or dabigatran. Normality was assessed visually using histograms and probability plots. Differences between categorical data were investigated using Pearson’s Chi squared test. Continuous data not normally distributed were analysed using two-sample Wilcoxon rank-sum (Mann–Whitney) test. Where data were normally distributed, Student’s *t* test was used. Regression analysis was done to investigate predictors of worse outcomes. Natural logarithm to transform non-parametric variables was done. Variables that were significantly different between groups were included in the regression model. To examine possible confounding and effect modification, we tested interactions between number of comorbidities and length of stay, polypharmacy and length of stay, atrial fibrillation and heart failure, number of comorbidities and renal failure and, finally, gender and renal failure. Missing values were dropped from analysis as the numbers were small representing less than 2.5% of the overall dataset. Significance threshold for all variables and interaction terms was set at a probability less than 0.05. All analyses were conducted using STATA version 15.

## Results

Data were analysed for 2307 patients. 200 patients (8.7%) were receiving an anticoagulant (warfarin or DOAC) at the time of admission. The majority were prescribed warfarin (167/200 patients, 83.5%) and atrial fibrillation was the most common indication (81.5%). Mean (SD) INR (International Normalised Ratio) of patients on warfarin preoperatively was 1.38 (0.2). Among patients on a DOAC, 22/33 patients (66.7%) were prescribed rivaroxaban and 11/33 patients (33.3%) prescribed apixaban. Patient characteristics were comparable between those anticoagulated and those not anticoagulated, except that the anticoagulated group had more men, a higher comorbid burden, less likely to be living with dementia, have a higher prevalence of cardiovascular disease, atrial fibrillation, polypharmacy, better cognition, and worse preoperative renal function (Table 1 in “Appendix").

**Table 1:** Characteristics of patients not on anticoagulation and patients on anticoagulation

Measure	Not on anticoagulation (*n* = 2107)	On anticoagulation (*n* = 200; 167 on warfarin, 33 on DOAC)	*p* value
Age			
Mean (SD), years	82.1 (9.9)	83.1 (7.6)	0.08
95% CI	81.7–82.5	82.0–84.2	
Gender			
Male, *n* (%)	594 (28)	75 (38)	0.01
Female, *n* (%)	1513 (72)	125 (63)	
Comorbidities			
Malignancy, *n* (%)	294 (14)	25 (13)	0.57
Dementia, *n* (%)	531 (25)	35 (18)	0.02
CVD, *n* (%)	1138 (54)	155 (78)	< 0.01
Diabetes Mellitus, *n* (%)	331 (16)	42 (21)	0.05
COPD, *n* (%)	275 (13)	32 (16)	0.24
Renal disease, *n* (%)	343 (16)	37 (19)	0.42
Hypertension, *n* (%)	931 (44)	87 (44)	0.85
Atrial fibrillation, *n* (%)	207 (10)	163 (82)	<0.01
Number of comorbidities			
Median (IQR)	2 (2)	3 (2)	< 0.01
Polypharmacy* *n* (%)	1150 (55)	152 (76)	< 0.01
Medication use			
Aspirin, *n* (%)	454 (22)	3 (2)	< 0.01
Clopidogrel, *n* (%)	167 (8)	2 (1)	< 0.01
ASA grade			< 0.01
1, *n* (%)	51 (2)	1 (0.50)	
2, *n* (%)	442 (21)	18 (9)	
3, *n* (%)	921 (44)	99 (50)	
4, *n* (%)	280 (13)	42 (21)	
5, *n* (%)	7 (0.33)	2 (1)	
Not stated, *n* (%)	406 (19)	38 (19)	
AMT score			< 0.01
0–6, *n* (%)	693 (33)	40 (20)	
7–10, *n* (%)	1366 (65)	154 (77)	
Not stated, *n* (%)	48 (2)	6 (3)	
Preoperative haemoglobin			
Mean (SD), g/dL	12.6 (5.5)	12.4 (2.0)	0.59
Preoperative eGFR			
Mean (SD), ml/min/1.73 m^2^	68.42 (20.83)	63.33 (20.80)	< 0.01

*SD* standard deviation, *CVD* cardiovascular disease, *COPD* chronic obstructive pulmonary disease, *IQR* interquartile range, *ASA* American Society of Anaesthesiologists, *AMT* abbreviated mental test, *eGFR* estimated glomerular filtration rate

*Four or more medications

Time to operation from admission to hospital was a median of 27 h in the anticoagulated group and 25 h in the non-anticoagulated group (*p* < 0.01). There was no difference in need for blood transfusion (Table 2 in “Appendix”). More from the anticoagulated group suffered postoperatively from heart failure (5.15% vs. 1.25%; *p* < 0.01) and renal failure (19% vs. 13.7%; *p* = 0.04). Otherwise, there were no differences in postoperative chest infection, urinary tract infection, haematoma and pressure ulcers (Table 2 in “Appendix”). Hospital mortality was similar in both groups (6.5% vs. 6.79%; *p* = 0.88). In the anticoagulated group, the median length of stay was 16 days and 14 days in the non-anticoagulated group (*p* < 0.01).

**Table 2:** Outcomes of patients not on anticoagulation and patients on anticoagulation

Measure	Not on anticoagulation (*n* = 2107)	On anticoagulation (*n* = 200)	*p* value
Time to operation			
Median (IQR), h	25 (15)	27 (17.5)	< 0.01
Blood transfusion			
Preoperatively, *n* (%)	71 (3)	7 (4)	0.92
Intra-operatively, *n* (%)	13 (1)	3 (2)	0.15
Postoperatively, *n* (%)	500 (24)	56 (28)	0.18
Length of stay			
Median (IQR), days	14 (10)	16 (12)	< 0.01
Health outcomes			
Heart failure, *n* (%)*	26 (1)	10 (5)	< 0.01
Renal failure, *n* (%)	288 (14)	38 (19)	0.04
Chest infection, *n* (%)	276 (13)	24 (12)	0.66
UTI, *n* (%)	208 (10)	20 (10)	0.95
Haematoma, *n* (%)	24 (1)	5 (3)	0.10
Pressure ulcer, *n* (%)	39 (2)	2 (1)	0.38
Serious wound or deep infection, *n* (%)	20 (1)	2 (1)	1.0
Stroke, *n* (%)	10 (0.5)	0 (0)	1.0
Myocardial infarction, *n* (%)	40 (2)	2 (1)	0.58
Hospital mortality, *n* (%)	143 (7)	13 (6.5)	0.88

*IQR* interquartile range, *UTI* urinary tract infection

*Missing data on 33 in the not anticoagulated group and 6 in the anticoagulated group

Length of stay was significantly longer, and the incidence of heart failure and renal failure was significantly higher in the anticoagulated group, but after controlling for confounders no longer became significant (Table 3 in “Appendix”).

**Table 3:** Linear regression analysis of length of stay, heart failure and renal failure

Statistical analysis	Coefficient	95% confidence interval	*p* value
Length of stay			
Crude estimate	1.14	1.05 to 1.24	< 0.01
Adjusted estimate*	1.04	0.94 to 1.14	0.49
Test for interaction			
Number of comorbidities	− 0.06	− 0.12 to 0.01	0.10
Polypharmacy	− 0.15	− 0.34 to 0.04	0.12
Heart failure			
Crude estimate	4.28	2.03 to 9.02	< 0.01
Adjusted estimate*	1.03	0.43 to 2.50	0.95
Test for interaction			
Atrial fibrillation	0.31	0.03 to 2.92	0.31
Renal failure			
Crude estimate	1.48	1.02 to 2.16	0.04
Adjusted estimate*	1.09	0.69 to 1.73	0.70
Test for interaction			
Number of comorbidities	1.08	0.78 to 1.48	0.65
Gender	1.08	0.50 to 2.31	0.85

*Adjusted for gender, dementia, atrial fibrillation, cardiovascular disease, number of comorbidities and polypharmacy

## Discussion

Hip fractures are a rapidly growing international public health problem. Many of the patients are older with multiple comorbidities of which some will require anticoagulation which will affect their hip fracture management. In this large cross-sectional analysis, the average patient with a hip fracture was in the ninth decade of life with at least two existing comorbid illnesses. There were differences in patient characteristics between those anticoagulated and those not anticoagulated. There were more men, a higher prevalence of cardiovascular disease, atrial fibrillation, worse renal function and better cognition in the anticoagulated group. Comparing these two groups’ outcomes, they were similar in relation to mortality, need for blood transfusion and postoperative complication, except the number of heart and renal failure. Length of stay appeared longer in the anticoagulated group. However, after adjusting for confounders, these postoperative complications and length of stay were no longer statistically different between the two groups.

Using a service registry to perform this cross-sectional analysis has limitations. Data reported were dependent on routine data collected as part of this registry. Information such as the prevalence of osteoporosis, comorbid burden (e.g. using the Charlson Comorbidity Index), haemoglobin levels, and complications such as delirium and thromboembolic event was not reported. In addition, the robustness of the data in this group of patients also very much depends on what is recorded by the patients’ clinical team. Besides that, the quality of the data entry may also affect the data accuracy. However, as the registry has been operational for almost 15 years, the local audit department has a wealth of experience to ensure data accuracy and missing data are minimised. This is seen in this analysis as the amount of missing data was less than 2.5% of a very large dataset capturing 3 years of patient level data. This study analysis was also limited by data that were only collected in this registry. Hence, there are likely confounding factors that we did not account for which might have affected the outcomes, for example sarcopenia and nutritional state. It was not possible to perform a subgroup analysis of patients on DOAC due to the low numbers.

To date, studies investigating the postoperative outcomes of patients admitted with hip fractures on anticoagulation have been mixed. One study found that neither time to surgery nor length of stay was significantly different when comparing those anticoagulated with warfarin and those not anticoagulated. Furthermore, they found no significant differences in thromboembolic event rates, bleeding complication, mortality, or 30-day readmission after surgery compared to those not taking warfarin on admission [20]. Another study reported that patient admitted on warfarin was associated with increased length of stay and lower survival at 12-months. Crucially, those anticoagulated had longer wait to surgery which can reach up to 46 h [16, 17]. The mixed picture could potentially be explained by the variability in how local practices differ in perioperative management of anticoagulation. To our knowledge, there are no prevailing national or international guidelines on how these patients should be managed and local consensus dictates clinical practice.

The biggest challenge in the management of patients with hip fracture on anticoagulation is how to safely deliver surgery. The advantage of warfarin is its ease in reversing its anticoagulation effect, it is possible to monitor its therapeutic effect, and being widely used mean clinicians have much more experience in addressing its anticoagulation effect in the peri-operative phase. DOACs lack that in clinical practice. Coagulation assays which have long been used to monitor drug anticoagulation effects are unreliable in DOACs. Anti-factor Xa levels can be checked to measure the effect of rivaroxaban, apixaban or edoxaban. Drug assay concentration is another option. However, these tests have their own limitation with reliability and standardised calibration. Hence, their use is not widespread and its place in routine clinical practice remains uncertain. Only one of the DOAC, dabigatran, has a specific reversal agent, idarucizumab. Its cost has restricted its widespread use. A recent study using audit data from a single hospital reported that hip fracture surgery for patients taking DOAC had a median time to theatre of 19 h [22]. Compared to a matched cohort, there was no difference in perioperative haemoglobin concentration, requirement for transfusion and reoperation suggesting that early surgery is possible even for patients on DOACs [22]. Our own study reported a median time to theatre of 27 h, which is within the required 36 h to achieve UK Best Practice Tariff for hip fracture management, a payment tariff for hospitals set nationally. Further research is clearly still required to better understand DOAC in the surgical setting [21].

## Conclusion

In this large cross-sectional analysis of a single university hospital’s trauma unit of people presenting with an acute hip fracture, we found that there was no significant difference in outcomes between those presenting on anticoagulated and those not. Prompt time to theatre can be safely delivered in those presenting on anticoagulation.

## Appendix

See Tables 1, 2, 3.
